# Application of multi-angle spaceborne observations in characterizing the long-term particulate organic carbon pollution in China

**DOI:** 10.21203/rs.3.rs-3734829/v1

**Published:** 2023-12-12

**Authors:** Yun Hang, Qiang Pu, Qiao Zhu, Xia Meng, Zhihao Jin, Fengchao Liang, Hezhong Tian, Tiantian Li, Tijian Wang, Junji Cao, Qingyan Fu, Sagnik Dey, Shenshen Li, Kan Huang, Haidong Kan, Xiaoming Shi, Yang Liu

**Affiliations:** a Gangarosa Department of Environmental Health, Rollins School of Public Health, Emory University, Atlanta, GA 30322, United States; b Department of Environmental and Occupational Health Sciences, School of Public Health, University of Texas Health Science Center at Houston, Houston, TX, 77030, United States; c School of Public Health, Fudan University, Shanghai 200032, China; d School of Public Health and Emergency Management, Southern University of Science and Technology, Shenzhen, 518055, China; e State Key Laboratory of Environmental Simulation and Pollution Control, School of Environment, Beijing Normal University, Beiji ng, 100875, China; f China CDC Key Laboratory of Environment and Population Health, National Institute of Environmental Health, Chinese Center for Disease Control and Prevention, Beijing, 100021, China; g School of Atmospheric Sciences, Nanjing University, Nanjing 210023, China; h Institute of Atmospheric Physics, Chinese Academy of Sciences, Beijing 100101, China; i State Ecologic Environmental Scientific Observation and Research Station at Dianshan Lake, Shanghai Environmental Monitoring Center, Shanghai 200235, China; j Centre for Atmospheric Sciences, Indian Institute of Technology Delhi, Hauz Khas, New Delhi 110016, India; k State Key Laboratory of Remote Sensing Science, Aerospace Information Research Institute, Chinese Academy of Sciences, Beijing, 100101, China; l Shanghai Key Laboratory of Atmospheric Particle Pollution and Prevention, Department of Environmental Science and Engineering, Fudan University, Shanghai 200433, China

**Keywords:** PM_2.5_ constituents, Organic carbon, MISR, Remote sensing, Fractional AOD, Super Learner

## Abstract

Ambient PM_2.5_ pollution is recognized as a leading environmental risk factor, causing significant mortality and morbidity in China. However, the specific contributions of individual PM_2.5_ constituents remain unclear, primarily due to the lack of a comprehensive ground monitoring network for constituents. This issue is particularly critical for carbonaceous species such as organic carbon (OC) and elemental carbon (EC), which are known for their significant health impacts, and understanding the OC/EC ratio is crucial for identifying pollution sources. To address this, we developed a Super Learner model integrating Multi-angle Imaging SpectroRadiometer (MISR) retrievals to predict daily OC concentrations across China from 2003 to 2019 at a 10-km spatial resolution. Our model demonstrates robust predictive accuracy, as evidenced by a random cross-validation R^2^ of 0.84 and an RMSE of 4.9 μg/m^3^, at the daily level. Although MISR is a polar-orbiting instrument, its fractional aerosol data make a significant contribution to the OC exposure model. We then use the model to explore the spatiotemporal distributions of OC and further calculate the EC/OC ratio in China. We compared regional pollution discrepancies and source contributions of carbonaceous pollution over three selected regions: Beijing-Tianjin-Hebei, Fenwei Plain, and Yunnan Province. Our model observes that OC levels are elevated in Northern China due to industrial operations and central heating during the heating season, while in Yunnan, OC pollution is mainly contributed by local forest fires during fire seasons. Additionally, we found that OC pollution in China is likely influenced by climate phenomena such as the El Niño-Southern Oscillation. Considering that climate change is increasing the severity of OC concentrations with more frequent fire events, and its influence on OC formation and dispersion, we suggest emphasizing the role of climate change in future OC pollution control policies. We believe this study will contribute to future epidemiological studies on OC, aiding in refining public health guidelines and enhancing air quality management in China.

## 1. Introduction

Ambient PM_2.5_ pollution, consisting of particulate matter with a diameter of 2.5 micrometers or less, has been identified as the leading environmental health risk factor in China (Liang et al., 2020). Over the past decade, the Chinese government has implemented strict air pollution control policies, effectively reducing total PM_2.5_ concentrations (Zhang et al., 2019). However, PM_2.5_ is defined by particle size rather than chemical composition, various components of PM_2.5_ have been found to individually affect human health in developed countries (Achilleos et al., 2017; Bell et al., 2007). A reduction in the concentration of total PM_2.5_ does not necessarily equate to a corresponding decrease in its health effects (Hang et al., 2022). The overall reduction in PM_2.5_ in China has been primarily due to decreased sulfate particles, yet the carbon components in PM_2.5_, such as elemental and organic carbon, may have elevated toxicity and are more challenging to control (Chowdhury et al., 2022; Hang et al., 2023; Liu et al., 2018; Yang et al., 2019). Both sulfate and organic carbon (OC) contribute significantly to the total PM_2.5_ concentration. However, while sulfate levels can be effectively reduced through desulfurization technologies, as demonstrated by the Chinese government (Meng et al., 2023), OC includes a wide range of organic compounds with both natural (i.e., biomass burning) and anthropogenic sources (i.e., vehicle exhausts, industrial processes). It can also be formed in the atmosphere from its precursors through secondary chemical reactions (i.e., oxidation of volatile organic compounds). In addition to OC’s direct impact on human health, it modulates incoming solar radiation and outgoing longwave radiation. Along with other aerosols and their interactions with clouds, OC influences the climate and climate change through its impact on Earth’s energy budget (Hang et al., 2019; Matus et al., 2019), thereby leading to profound impacts on our living environment and society.

Although air pollution in China has seen major improvements, the country’s rapid industrialization, urbanization, and increasing vehicular traffic still place it among the most polluted countries in the world. Given its large and aging population, monitoring PM_2.5_ constituents is crucial for identifying pollution sources, formulating targeted air pollution control policies to protect public health. Traditionally, networks of ground monitoring stations have been the primary method for measuring air pollution. China has established extensive monitoring sites across the nation to track total PM_2.5_ levels. However, the sites for PM_2.5_ constituents were established later than those for total PM_2.5_ and are sparsely distributed. It results in China facing constraints in resources to comprehensively observe nationwide PM_2.5_ constituents and their spatiotemporal variations. To evaluate historical OC patterns and their long-term health effects in China without a dense monitoring network, several studies have turned to chemical transport models (CTMs) (Liu et al., 2022). But the accuracy of these models depends heavily on precise initial and boundary conditions. Predicting OC is especially difficult due to its connection with secondary organic aerosol (SOA) formation, introducing additional uncertainties because of the complex chemical processes and varied precursors involved (Pöschl, 2005; Weber et al., 2007). Another significant challenge is the reliance of CTMs on up-to-date and accurate emission inventories, which are often lacking in developing countries like China. The latest nationwide OC prediction model by Liu et al., (2022) utilized the Weather Research and Forecasting–Community Multiscale Air Quality modeling system. The model achieved a correlation coefficient of 0.72 when comparing observed and simulated daily mean OC across China from 2013 to 2021, with a spatial resolution of 10-km.

Satellite remote sensing of aerosol optical depth (AOD) has emerged as a powerful tool to bridge the gap in monitoring ground-level air pollution in data-scarce regions. It can be used to develop large-scale and long-term PM_2.5_ models based on the PM_2.5_-AOD relationship (Ma et al., 2022). Many studies have utilized AOD data retrieved from the Moderate Resolution Imaging Spectroradiometer (MODIS) instruments aboard NASA’s Terra and Aqua satellites to estimate PM_2.5_ levels in China. The most recent OC model developed by Liu et al. (2022) also employed such satellite data. Although MODIS AOD has been successfully used to predict total PM_2.5_ levels, it may introduce large biases in estimating PM_2.5_ constituents (Liu et al., 2007b). This is because MODIS instruments are single-angle, polar-orbiting passive sensors that provide limited information on aerosol optical characteristics under clear sky conditions. However, estimating PM_2.5_ constituents requires comprehensive information on particle shape and their distinct radiative effects to distinguish individual aerosol types. NASA’s Multi-angle Imaging SpectroRadiometer (MISR) aboard the Terra satellite, which was launched in December 1999, offers a unique capability in this regard. MISR is the only instrument available that can monitor historical aerosol levels in China from a multi-angled perspective, providing total AOD as well as AOD fractionated by shape, size, and light-absorbing properties. This aids in identifying the complex spatiotemporal variability of PM_2.5_ constituents (Franklin et al., 2017; Hang et al., 2022). Although MISR is also a passive sensor, its nine cameras observe from different viewing angles along the satellite’s path in four spectral bands, enabling a more robust separation of the Earth’s surface features from the atmospheric layer (Diner et al., 1998; Kahn et al., 2005).

In recent years, machine learning has been widely applied to further exploit the use of massive satellite data for studying air pollution (Xiao et al., 2018). In this study, we trained ensemble machine learning algorithms, specifically the Super Learner, to fully explore MISR’s data capabilities in reconstructing historical OC concentrations across China from 2003 to 2019, with a spatial resolution of 10 km. Moreover, in conjunction with our previous long-term elemental carbon (EC) model in China (Hang et al., 2023), we calculated the OC/EC ratio, an important metric for understanding PM_2.5_ pollution sources and characteristics. Our results will not only help evaluate the efficacy of existing air pollution policies in China but will also benefit future epidemiological studies on the health effects of OC, and have implications for climate and climate change research.

## 2. Study design and data

### 2.1. Study domain

China is the world’s second-most populous country, with a population exceeding 1.4 billion. It was among the most air-polluted countries in the world, and the current PM_2.5_ concentration level is still much higher than WHO recommendations. To support future epidemiological investigations on the long-term health effects of OC in China, our model estimates daily OC concentrations across the entire country, including all provinces, autonomous regions, and special administrative regions. The modeling grid, at a 10 × 10 km^2^ spatial resolution, includes 100,699 grid cells across China. Fig. S1 shows the study domain and the location of available OC monitoring sites involved in the model development. The modeling period is 2003–2019 which covers the years before the 11^th^ Five-Year Plan (FYP, 2006–2010), the first FYP that emphasized environmental protection and marked a shift towards sustainable development in China. It also captures the extreme peak of air pollution in China in 2013 and stretches to 2019, including the implementation period of the strictest air policy ever, the Air Pollution Prevention and Control Action Plan (APPCAP, 2013–2017), and the years before COVID-19.

We selected three study areas to investigate the regional pollution discrepancies of OC, as highlighted in Fig. S1. First, the Beijing-Tianjin-Hebei region (BTH), where the capital Beijing is located was the most polluted area, especially during the extreme air pollution event in 2013. Second, we focused on the Fenwei Plain (FWP), which consists of Shaanxi, Shanxi, and Henan provinces, and has experienced significant PM_2.5_ pollution due to a reliance on coal in power production, industrial activities, and residential heating, particularly during winter months. Third, we chose Yunnan, a less industrialized province with a complex landscape of mountains, plateaus, and diverse plant life. Biomass burning in rural areas, forest fires during dry seasons, and agricultural burning from neighboring countries are significant contributors to air pollution in Yunnan.

### 2.2. Ground OC data

Approximately 18,500 daily ground OC measurements were collected from 46 monitoring sites across China to develop the model, including sites in Taiwan and Hong Kong. Although a regulatory PM_2.5_ speciation network does not exist in China, the available ground OC data have covered urban and rural regions with different population densities (Hang et al., 2022). These OC measurements are mainly derived from a standard method equipped with PM_2.5_ samplers using quartz filters. Prior to sampling, these quartz filters are preheated at 450°C for several hours to eliminate any organic compounds. After the sampling phase, the filters are analyzed using the thermal-optical reflectance method with the Desert Research Institute OC/EC analyzer, which is commonly used in atmospheric chemistry and air quality studies. The filter analysis follows the IMPROVE protocol as described in Chow et al., (2007). Details about these measurements and data sources are provided in Table S1. Note that mean daily OC values were calculated if multiple ground monitors fell within a single 10-km model grid cell.

### 2.3. MISR fractional AOD data

MISR can measure reflected sunlight at nine distinct viewing angles (0°, ±26.1°, ±45.6°, ±60°, ±70.5°) across four spectral bands (446 nm, 558 nm, 672 nm, 866 nm). This unique multi-angle and multi-spectrum design enables space-borne observations of multiple aerosol types and microphysical properties to estimate ground-level PM_2.5_ constituent concentrations over intricate land surfaces (Diner et al., 1998; Liu et al., 2007b). Here, we used the most recent MISR Level 2 Version 23 aerosol products from NASA’s Langley Research Center Atmospheric Science Data Center. The new MISR products offer an enhanced spatial resolution of 4.4 × 4.4 km^2^ and superior accuracy through a series of algorithmic advancements, including radiometric correction and additional cloud screening (Garay et al., 2020). We applied this MISR data product to calculate fractional AODs with 74 aerosol mixtures from MISR retrievals according to the methods documented in Liu et al. (2007b). Considering the microphysical properties of OC, a list of fractional AODs including #6, #8, and #21 were selected as predictors to train our model. In this study, we calculated and aggregated the annual mean MISR data into the 10-km modeling grid, given that MISR’s swath is relatively narrow.

### 2.4. Other satellite data and supporting information

Satellite remote sensing offers unparalleled insights into both anthropogenic and natural factors contributing to OC pollution. In our study, we enhanced the OC model by incorporating data on eight land cover types such as shrubland, cropland, and built-up land. These were sourced from the C3S global land cover products (https://www.esa-landcover-cci.org/), available at a 300-meter resolution and produced by the European Space Agency’s Climate Change Initiative. To account for the effect of cloud cover on MISR AOD retrievals, we integrated the Clouds and the Earth’s Radiant Energy System (CERES) monthly cloud fraction (CF) data into our model, with a spatial resolution of 1° × 1°. To match the modeling grid, we resampled the CF data to a 10 km × 10 km resolution using the inverse distance weighting (IDW) method. Additionally, we incorporated annual population count data from WorldPop (https://www.worldpop.org/) at a 100-meter resolution and elevation data from the Global Multi-resolution Terrain Elevation Data 2010 (GMTED 2010), which offers a 30-arc second resolution DEM (approximately 1 km) and is derived from multiple satellite sources. Land cover type, population, and elevation data were aggregated to align with the OC modeling grid. Moreover, we integrated high temporal resolution atmospheric reanalysis data to increase the model’s performance. Daily simulated OC concentration at 0.5° × 0.625° resolution was obtained from the Modern-Era Retrospective Analysis for Research and Applications model (MERRA-2) (Randles et al., 2017). Considering potential meteorological conditions affecting OC formation, daily temperature and humidity at 2 meters, and the planetary boundary layer height (PBLH) were downloaded from the Goddard Earth Observing System Model at a resolution of 0.5° × 0.625° (2003–2012) and a resolution of 0.25° × 0.3125° (2013–2019). All these data have been resampled to the OC model’s resolution using the IDW method.

## 3. Modeling approach and methods

We utilized the Super Learner (SL), an ensemble method in machine learning, to predict daily OC concentrations across China from 2003 to 2019. The SL aims to mitigate overfitting and improve the prediction accuracy of traditional machine learning algorithms. It can achieve the best overall prediction performance by formulating a weighted ensemble of individual machine learning algorithms (a.k.a. base learners) that collectively minimize the cross-validated error (Van der Laan et al., 2007). Although SL accommodates diverse base learner algorithms, we included only those established ones that have been widely applied in PM_2.5_ modeling to limit the uncertainties of selecting base learners. Our OC model was optimally built by the weighted sum of predictions from three base learners, which includes Random Forest (RF), Extreme Gradient Boosting (XGB), and LightGBM (LGB), using a non-negative least squares model (Phillips et al., 2022). Because previous studies showed that machine learning-based models are prone to underestimation for extreme pollution events (Ma et al., 2022; Xiao et al., 2018), we applied the Synthetic Minority Over-sampling Technique (SMOTE) to oversample the underrepresented OC extremes (Vu et al., 2022). Given the original training data, SMOTE generates synthetic samples using information from the original underrepresented samples and their nearest five neighbors (Blagus and Lusa, 2013; Chawla et al., 2002). We used SMOTE to oversample OC concentrations over 37.5 and below 55 μg/m^3^ (97^th^ and 99^th^ percentiles) once (Zhang et al., 2023). The oversampled data accounted for 3.6% of the training dataset, ensuring the oversampling did not skew the distribution of OC concentrations.

The two equations below summarize the definition of the OC prediction model:

(1) $$OC_{i,j}=f_{m}\left(AOD_{i,j},X_{i,j},Z_{i}\right),m=1,2,3$$

(2) $${\overset{ˆ}{f}}_{SL}={\overset{ˆ}{f}}_{NNLS}({\hat{OC}}_{i,j})={\overset{ˆ}{a}}_{1}{\hat{OC}}_{RF,i,j}+{\overset{ˆ}{a}}_{2}{\hat{OC}}_{XGB,i,j}+{\hat{a}}_{3}{\hat{OC}}_{LGB,i,j}$$

Where $OC_{i,j}$ represents the estimated ground-level OC concentrations using a base learner model $f_{m}$ at grid cell i on day j. Here, m=1,2,3 corresponds to the three base learners: RF, XGB, and LGB, respectively. $AOD_{i,j}$ denotes the corresponding collection of MISR fractional AODs. $X_{i,j}$ is a set of spatiotemporal covariates, such as meteorological conditions, cloud fraction, OC simulations, population introduced in the data section. $Z_{i}$ is a vector of temporally invariant covariates, including elevation and land type. ${\overset{ˆ}{f}}_{SL}$ represents the OC predictions using SL, which is based on the non–negative least squares (NNLS) model (Phillips et al., 2022), denoted as ${\overset{ˆ}{f}}_{NNLS}({\hat{OC}}_{i,j})$. The final model was determined by the weighted sum of predictions from three base learners.

We employed a 10-fold cross-validation (CV) technique for OC model development and validation (Shtein et al., 2019). Initially, we randomly divided the training data into 10 subsets. Of these, 9 subsets were used for model development, and the remaining subset was used to validate the model predictions. This process was iteratively repeated 10 times, ensuring each subset served as the testing set. Specifically, within the training set, we applied a nested CV strategy to construct the SL-based OC prediction model (Cawley and Talbot, 2010). This involved two 10-fold CV loops: the inner CV loop for fine-tuning the hyperparameters of the base learner algorithms, and the outer CV loop for obtaining optimal weighted SL predictions. To assess the robustness and detect potential overfitting in space and time, we also conducted spatial and temporal CVs. Unlike the random 10-fold CV, the spatial CV created a testing set by randomly selecting 10% of the modeling grid cells. A leave-one-year-out temporal CV was used, where each year’s data served as the testing set. For enhanced model interpretation, we calculated the variable importance of each predictor by quantifying its permutational improvement in mean squared error. We evaluated the model performances using the CV R^2^ and root mean squared errors (RMSE) across all CV settings.

## 4. Results and discussion

### 4.1. MISR-driven OC exposure model

**Fig. 1.**
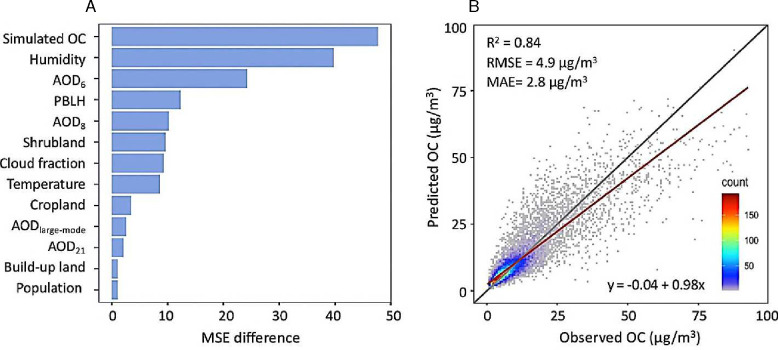
(A) Variable importance of top predictors in the OC model based on their contributions to reducing the mean squared error (MSE, with MSE > 1.0). (B) Linear regression comparing observed and predicted daily OC concentrations (unit: μg/m^3^). The R^2^ value is derived from a 10-fold random cross-validation assessing the model’s performance. The red line shows the regression line, while the dark grey line represents the 1:1 line.

Fig. 1A illustrates the variable importance of top predictors in the OC model, based on their contributions to reducing mean squared errors. MERRA-2 simulated OC level ranks at the top. This indicates MERRA-2’s ability to assist in regional air quality modeling, although its spatial resolution (0.5° × 0.625°) is much coarser than the OC model’s resolution (about 0.1° × 0.1°). This provides an opportunity for regions lacking sufficient resources on satellite remote sensing of their environment. Humidity is the second most important predictor, given its crucial role in the formation and growth of SOAs. For instance, a high-humidity environment promotes the hydrolysis and oxidation processes that transform volatile organic compounds into SOAs. Additionally, existing OC particles can absorb more moisture from the environment, increasing their particle size and altering their optical properties. MISR fractional AOD #6 that represents spherical and non-absorbing particles, ranks third in the model. We integrated the MISR data at an annual mean level into the OC model due to its limited temporal resolution. In addition, land cover type information is critical to the model. For instance, information on shrubs and croplands is among the top 10 contributors to the model, likely due to their association with emissions from biomass burning and biogenic sources (Chow et al., 2011). Note that more than one-third of the model’s predictors directly utilize data retrieved from satellite instruments (i.e., MISR, CERES), and the remaining predictors, although based on model simulations (i.e., MERRA-2, GEOS, etc.), were developed by simulating satellite data. This demonstrates that satellite remote sensing is essential in developing modern air pollution models.

Fig. 1B shows the 10-fold cross-validation (CV) results of our daily OC prediction model that uses SL integrating available ground data, MISR fractional AODs, atmospheric reanalysis, and supporting information. The model exhibits high prediction performance with an overall random CV-R^2^ of 0.84 and a CV-RMSE of 4.9 μg/m^3^, an improvement over using RF, XGB, or LGB alone. Although the ground monitoring data for OC were relatively sparse in space and time, our SL-based model demonstrates great performance in both spatial and temporal 10-fold CVs (Table S2). Fig. S2 shows that our model predictions accurately captured the temporal variability of OC across China under varying geographic and pollution conditions. The overall predicted daily mean OC concentration was 10.4 μg/m^3^, as compared to 10.1 μg/m^3^ calculated from ground observations. WHO’s air quality guideline states that the 24-hour total PM_2.5_ mass concentration should not exceed 15 μg/m^3^ for more than 3–4 days per year, indicating the imperative need to control OC pollution to protect public health in China. Compared with previous studies conducted at national scales, our model’s CV-R^2^ of 0.84 is higher than the out-of-bag R^2^ of 0.71 for the OC model built for the United States using RF (Di et al., 2016). The Pearson correlation coefficient (r) between observed and predicted values at the daily level was 0.88 in this study, a significant improvement over the most recent OC model for China with an r of 0.67 (Liu et al., 2022). Our robust OC model performance can be attributed to three main reasons: first, the unique aerosol information provided by MISR fractional AODs (e.g., shape, size, and extinction), which aids in capturing characteristics of particulate OC (Liu et al., 2007a); second, the SL’s ability to reduce the unexplained bias of individual machine learning algorithms such as RF; and third, models that rely heavily on CTM simulations may be subject to high uncertainty, likely propagated from uncertain emission inventories and complex chemical reactions in the atmosphere.

### 4.2. Spatiotemporal distributions of OC in China

**Fig. 2.**
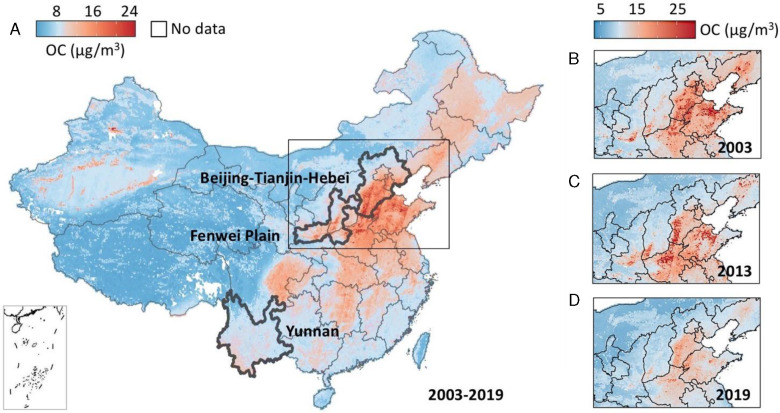
(A) Nationwide annual mean OC concentrations across China from 2003 to 2019. (B)-(D) Regional annual mean OC concentrations for 2003, 2013, and 2019, respectively. Hereafter, all concentration units are in μg/m^3^.

Fig. 2A illustrates the geographical distribution of predicted annual mean OC concentrations across China over the 17-year study period. Elevated OC levels were predominantly found in the plains of northern, central, and southwestern China. Notably, in some areas of the BTH and FWP regions, annual mean OC can surpass 20 μg/m^3^, which is likely attributable to combined emissions from vehicular traffic, industrial operations, central heating, and agricultural activities. Conversely, the coastal areas of southeastern China, including Hainan and Taiwan, as well as the western regions, exhibit comparatively lower OC levels, generally under 8 μg/m^3^. These spatial patterns in OC pollution have remained consistent over the years; however, a notable decrease was observed in the Northern China Plain from 2003 to 2019, as depicted in Figs. 2B–2D. A significant shift in OC levels is evident in the 2013 map, benefitting from a list of control policies starting from the 11^th^ FYP to APPCAP, which significantly reduced air pollution in urban and industrialized areas (Zhang et al., 2019). The contrast in OC distribution between urban and rural areas outside the heavily polluted North China Plain, particularly in the southern provinces, was less pronounced. This distribution was linked to rapid urbanization in rural locales and the migration of industrial facilities from urban to rural settings (Feng et al., 2006). Additionally, our model suggests that fluctuations in OC pollution in China might be influenced by climate phenomena such as the El Niño-Southern Oscillation (ENSO) (Sun et al., 2018). For instance, during the El Niño periods of 2014/2015 and 2018/2019, BTH experienced annual mean OC concentrations that were 1.9 μg/m^3^ and 0.9 μg/m^3^ higher, respectively, than those during the 2017/2018 La Niña period, despite decreasing emissions being reported in the region over the years. A possible reason is that El Niño brings warmer and drier conditions to the BTH, which can increase the risk of wildfires that cause OC pollution, and at the same time, reduce precipitation (Zhao et al., 2022). This reduction in precipitation impedes the removal of OC pollution within the boundary layer. Conversely, La Niña is associated with wetter weather conditions that increase precipitation, thereby increasing wet deposition.

**Fig. 3.**
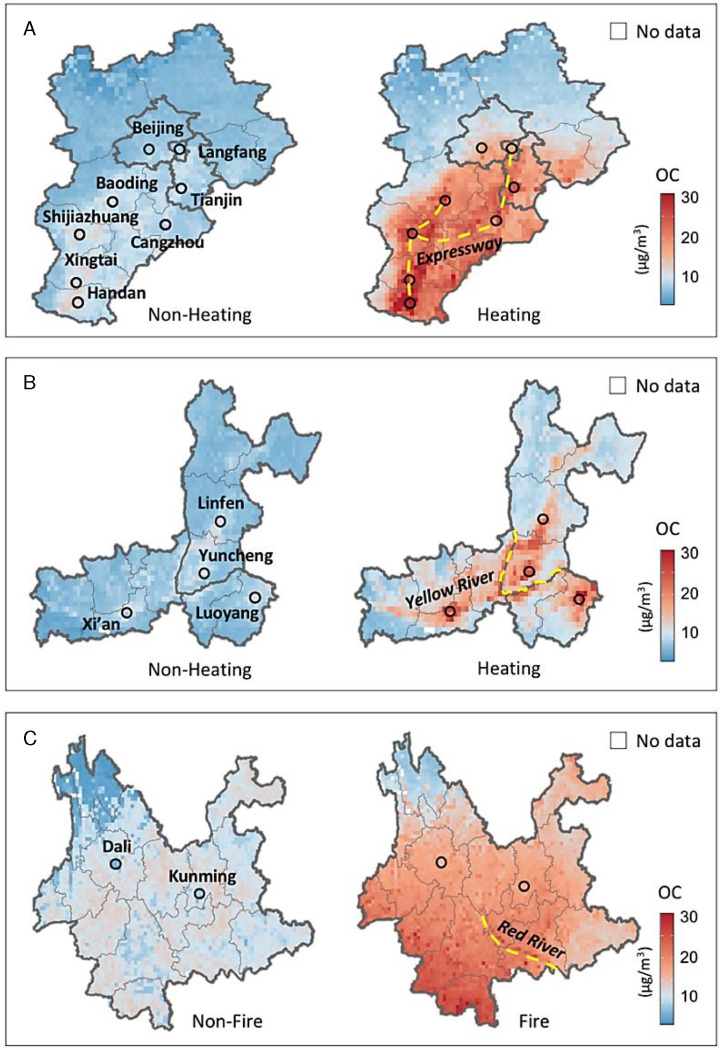
(A) 17-year annual mean OC concentrations in the Beijing-Tianjin-Hebei region from 2003 to 2019, comparing non-heating and heating seasons. (B) Similar to (A), but for the Fenwei Plain. (C) Similar to (A), but for Yunnan Province, comparing non-fire and fire seasons.

We further compared the spatiotemporal distribution patterns of OC pollution across the three selected regions: BTH, FWP, and Yunnan, as shown in Fig. 3. In BTH and FWP, OC concentrations during the heating season (December–February) were much higher than those in the non-heating season (June–August). We observed an increase in annual mean OC from 9.0 μg/m^3^ to 14.9 μg/m^3^ in BTH, and from 8.6 μg/m^3^ to 13.0 μg/m^3^ in FWP, transitioning from the non-heating to heating seasons (Figs. 3A–3B). The elevated OC levels during the heating season are primarily due to anthropogenic emissions from fossil fuel combustion and biomass burning for domestic heating (Liu et al., 2018). Additionally, vehicular emissions along major traffic corridors contribute to OC pollution, as depicted by the yellow dashed lines in Fig. 3A. Elevated OC levels were observed along the Yellow River in FWP (yellow dashed line in Fig. 3B), likely modulated by dam operation (Lu et al., 2022). Regarding natural impact factors, the stagnant meteorological conditions with weak winds and low boundary layer height in winter also promote the accumulation of particulate OC.

Our unique MISR-driven model revealed distinctive characteristics of OC pollution in Yunnan Province (Fig. 3C). During the fire seasons (February–April), intense OC pollution was primarily contributed by agricultural biomass burning (Wang et al., 2023). Southern Yunnan experienced the most severe impact, with an average OC concentration of 11.3 μg/m^3^. Conversely, during the non-fire seasons (August–October), OC concentrations were considerably lower and more uniformly distributed, with an average of 7.4 μg/m^3^. Moreover, OC pollution in Yunnan is influenced by the long-range transport of biomass burning from neighboring Southeast Asian countries (Yang et al., 2020). We observed a pronounced OC concentration gradient from south to north in Yunnan, aligning with the dominant southwesterly winds during the fire season. Notably, our model observes increased OC levels over county towns along the Red River Valley, where limited air circulation results in annual mean OC concentrations being 1–2 μg/m^3^ higher than in the surrounding mountainous areas (highlighted as yellow dashed lines in Fig. 3C). These findings suggest that our satellite observation-driven model can effectively capture the fine-scale, complex distribution of OC pollution influenced by local geographical conditions across China. Such OC pollution characteristics were not captured in coarser resolution atmospheric reanalysis such as MERRA-2 or in heavily CTM-based air quality models (Liu et al., 2022).

### 4.3. Source contributions of carbonaceous pollution

Using previously published EC exposure model predictions (Hang et al., 2023), we calculated the OC/EC ratio to explore the source of carbonaceous air pollution in China. This ratio has been frequently used to diagnose the relative contributions of primary particulate matter emissions and SOAs formed from precursors. This is because EC originates from primary emissions, while OC comes from both primary emissions and secondary formations (Rogge et al., 1993). Higher OC/EC ratios indicate a greater contribution from SOA formation or biomass burning, while lower ratios suggest fossil fuel combustion in vehicular exhaust and industrial processes (Gu et al., 2010; Pio et al., 2011; Turpin and Lim, 2001; Zeng and Wang, 2011; Zhang et al., 2020).

**Fig. 4.**
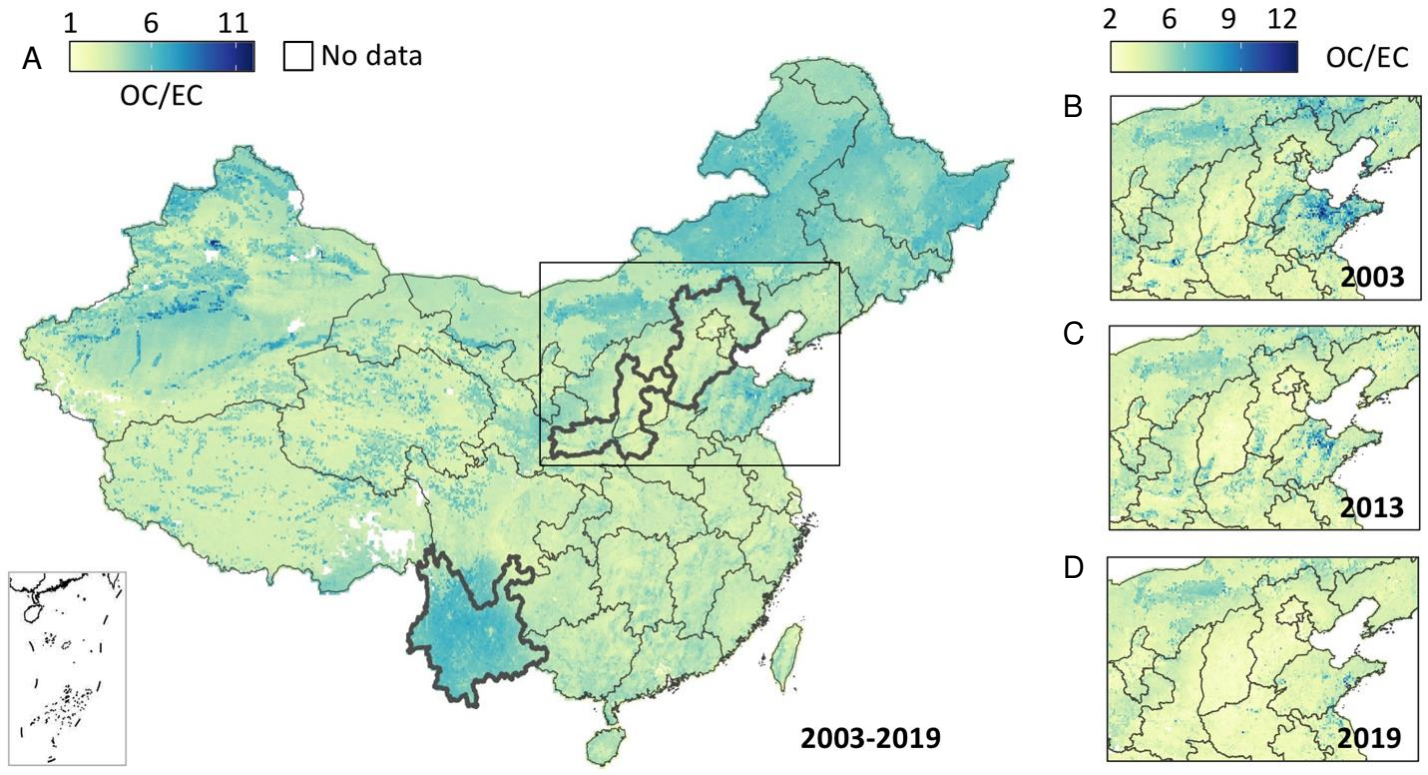
Similar to Fig. 2 but for OC/EC ratios.

The annual mean OC/EC ratio across China from 2003–2019, and in selected years, is shown in Fig. 4. Unlike the spatial patterns of OC concentrations (Fig. 2), higher OC/EC ratios were observed in the less-populated rural regions such as northeastern and southwestern China, indicating a strong influence from agricultural and biogenic emissions. In contrast, lower OC/EC ratios appeared in populous city clusters, suggesting a high impact from primary pollution sources. Since 2013, we have noticed a significant ratio decrease in the North China Plain, where sources from biomass burning have been substantially reduced due to recent air pollution control policies (Ma et al., 2019). However, the ratio over less industrialized regions like Yunnan remained constant over the years.

**Fig. 5.**
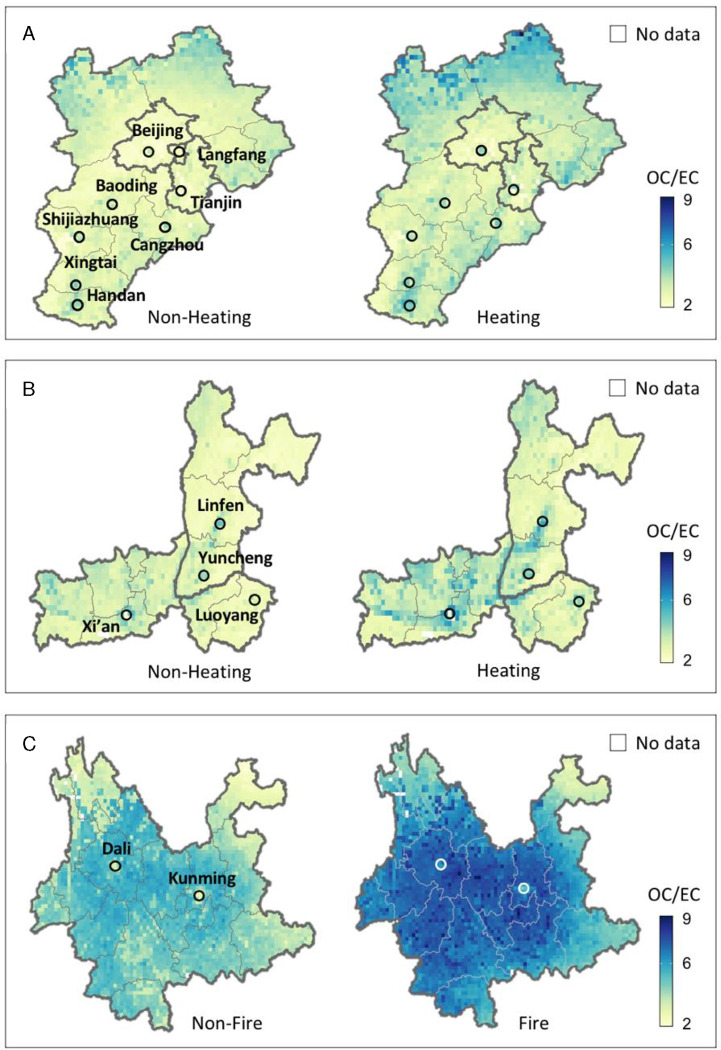
Similar to Fig. 3 but for OC/EC ratios.

A comparison of the regional OC/EC distribution discrepancy among BTH, FWP, and Yunnan is shown in Fig. 5. We found that the overall annual mean OC/EC ratio during the heating season was higher than that during the non-heating season near major cities in northern China, especially in FWP (Figs. 5A and 5B). A possible reason is fugitive emissions and the use of biomass in unregulated devices for winter heating. Additionally, wintertime meteorological conditions such as a lower boundary layer height and frequent inversions enhance the formation of secondary OC through absorption and condensation of semi-volatile organic compounds by atmospheric particles (Duan et al., 2020; Gu et al., 2010). Interestingly, our model observed a consistent and relatively low OC/EC ratio in Beijing than in adjacent provinces (Fig. 3A). This finding suggests that the adjustment of the energy structure, such as shifting from coal to natural gas, has effectively reduced OC pollution in Beijing (Liu et al., 2018). In Yunnan, the OC/EC ratio was significantly higher during the fire season than in the non-fire season (Fig. 5C). Given that industrial sources are scarce in this region, the OC pollution likely mainly originated from biomass burning and VOCs emitted by trees and other vegetation. Recent studies indicate that Yunnan, known for the highest incidence of forest fires in China, is witnessing an escalation in extreme particulate pollution events during fire seasons, primarily due to local forest fires (Yang et al., 2022). Climate change is contributing to an increased frequency and expanded range of forest fires around the world, significantly threatening human health (Romanello et al., 2023). We suggest that for further controlling air pollution in China, climate change is a factor that needs to be considered in future air policy planning.

## 5. Conclusion

Our study is the first to incorporate MISR fractional AOD data for modeling long-term OC exposure in China. Despite the absence of a ground monitoring network for PM_2.5_ constituents, MISR data can successfully be used to predict daily OC concentrations across the entire country. Additionally, the implementation of an ensemble machine learning algorithm significantly enhanced our model’s performance, as evidenced by improved R^2^ and RMSE values. Predictions from our model will be applied in epidemiological studies investigating the long-term health effects of OC exposure in China. Furthermore, our study is the first to examine the characteristics of carbonaceous particulates across continuous spatial and temporal dimensions at a national scale in China, providing necessary information to evaluate the effectiveness of existing air quality policies.

## Highlights

- Incorporating MISR AOD data, our model (R^2^ = 0.84) can hindcast daily particulate organic carbon in China.
- The application of Super Learner leverages the strengths of various algorithms to predict organic carbon concentrations.
- We suggest considering climate change as a key factor in controlling organic carbon pollution in China.
